# Supplementary material for: MetaRibo-Seq measures translation in microbiomes
Source: Nat Commun. 2020 Jun 29;11:3268. doi: 10.1038/s41467-020-17081-z (PMC7324362; doi:10.1038/s41467-020-17081-z)
Supplement: Supplementary file 10 — Supplementary Data 7 [file 41467_2020_17081_MOESM10_ESM.zip › File2/Confidence_VeryHigh_Taxonomy/347194_out.krona.html]

Javascript must be enabled to view this page.

members
magnitude
magnitudeUnassigned
count
unassigned
taxon
rank

347194\_out

21

21
superkingdom
2

976
21
phylum

21
class
200643

order
21
171549

family
20
815

816
20
genus

821

SRS018351\_contig\_number\_16946SRS051610\_contig\_number\_9984SRS075984\_contig\_number\_27506
species
3


SRS014613\_contig\_number\_15352SRS064276\_contig\_number\_30228
820
species
2

6
species
1907658

SRS016335\_contig\_number\_29540SRS047014\_contig\_number\_32877SRS100907\_contig\_number\_8686SRS1041031\_contig\_number\_contig-100\_1983.143928SRS147088\_contig\_number\_contig-100\_7.51833SRS149879\_contig\_number\_20117

species
2

SRS142542\_contig\_number\_3077SRS148817\_contig\_number\_13219
246787

1262747

SRS893341\_contig\_number\_9654
species
1

818

SRS048981\_contig\_number\_1200SRS075021\_contig\_number\_1058
2
species

817

SRS142890\_contig\_number\_23139
species
1


SRS050974\_contig\_number\_contig-100\_80.24119SRS056273\_contig\_number\_5522SRS075078\_contig\_number\_15927
387090
species
3

2005519
1
family

1
genus
397864

2033407

SRS1041140\_contig\_number\_21378
1
species
